# Correction: Finkelstein et al. How Can Ice Emerge at 0 °C? *Biomolecules* 2022, *12*, 981

**DOI:** 10.3390/biom13121687

**Published:** 2023-11-23

**Authors:** Alexei V. Finkelstein, Sergiy O. Garbuzynskiy, Bogdan S. Melnik

**Affiliations:** 1 Institute of Protein Research, Russian Academy of Sciences, 142290 Pushchino, Russia; sergey@phys.protres.ru (S.O.G.); bmelnik@phys.protres.ru (B.S.M.); 2 Faculty of Biotechnology, Lomonosov Moscow State University, 142290 Pushchino, Russia; 3 Faculty of Biology, Lomonosov Moscow State University, 119192 Moscow, Russia

We regret to state that our article “How Can Ice Emerge at 0 °C?” [1] contains an annoying bug—an error in the sign of the exponent in Equation (6)—leading to some other bugs (that do not change the main results of the work, but are still annoying); they are listed and corrected below. The modified places are in bold.

(1) Page 4, Equation (6): Error in the sign of the exponent for *n*_0_;

should be:

$n_{0}^{\frac{3-d}{2d}}$.

(2) Page 4, the first line below the Equation (6) should be:

so $\tau \sim 0.5\cdot 10^{-7}$ s at d=3 and $\tau \sim 0.7\cdot 10^{-7}n_{0}^{\frac{1}{4}}\approx 0.3\cdot 10^{-7}\sqrt{100^{o}/∆T}$ s at d=2.

(3) Page 5, in the formula in the sixth line of Section 2.3. *The Time of Ice Growth after the Nucleation* should be:

$$\tau_{1}\left(\frac{k_{B}T}{-∆\mu }\right)\approx 10^{-7}s\cdot \left(\frac{100^{o}}{∆T}\right)$$

(4) Page 7, in the Equation (11) should be:

$$\sim \frac{0.3\cdot 10^{-7}\sqrt{100^{o}/∆T}}{N_{S}}\cdot exp\left(\frac{260^{o}}{∆T}\right)\;s.$$
